# Digital image processing techniques for detecting, quantifying and classifying plant diseases

**DOI:** 10.1186/2193-1801-2-660

**Published:** 2013-12-07

**Authors:** Jayme Garcia Arnal Barbedo

**Affiliations:** Embrapa Agricultural Informatics, Campinas, SP Brazil

## Abstract

This paper presents a survey on methods that use digital image processing techniques to detect, quantify and classify plant diseases from digital images in the visible spectrum. Although disease symptoms can manifest in any part of the plant, only methods that explore visible symptoms in leaves and stems were considered. This was done for two main reasons: to limit the length of the paper and because methods dealing with roots, seeds and fruits have some peculiarities that would warrant a specific survey. The selected proposals are divided into three classes according to their objective: detection, severity quantification, and classification. Each of those classes, in turn, are subdivided according to the main technical solution used in the algorithm. This paper is expected to be useful to researchers working both on vegetable pathology and pattern recognition, providing a comprehensive and accessible overview of this important field of research.

## Introduction

Agriculture has become much more than simply a means to feed ever growing populations. Plants have become an important source of energy, and are a fundamental piece in the puzzle to solve the problem of global warming. There are several diseases that affect plants with the potential to cause devastating economical, social and ecological losses. In this context, diagnosing diseases in an accurate and timely way is of the utmost importance.

There are several ways to detect plant pathologies. Some diseases do not have any visible symptoms associated, or those appear only when it is too late to act. In those cases, normally some kind of sophisticated analysis, usually by means of powerful microscopes, is necessary. In other cases, the signs can only be detected in parts of the electromagnetic spectrum that are not visible to humans. A common approach in this case is the use of remote sensing techniques that explore multi and hyperspectral image captures. The methods that adopt this approach often employ digital image processing tools to achieve their goals. However, due to their many peculiarities and to the extent of the literature on the subject, they will not be treated in this paper. A large amount of information on the subject can be found in the papers by Bock et al. (2010), Mahlein et al. (2012) and Sankaran et al. (2010).

Most diseases, however, generate some kind of manifestation in the visible spectrum. In the vast majority of the cases, the diagnosis, or at least a first guess about the disease, is performed visually by humans. Trained raters may be efficient in recognizing and quantifying diseases, however they have associated some disadvantages that may harm the efforts in many cases. Bock et al. (2010) list some of those disadvantages:

- Raters may tire and lose concentration, thus decreasing their accuracy.
- There can be substantial inter- and intra-rater variability (subjectivity).
- There is a need to develop standard area diagrams to aide assessment.
- Training may need to be repeated to maintain quality. Raters are expensive.
- Visual rating can be destructive if samples are collected in the field for assessment later in the laboratory.
- Raters are prone to various illusions (for example, lesion number/size and area infected).

Besides those disadvantages, it is important to consider that some crops may extend for extremely large areas, making monitoring a challenging task.

Depending on the application, many of those problems may be solved, or at least reduced, by the use of digital images combined with some kind of image processing and, in some cases, pattern recognition and automatic classification tools. Many systems have been proposed in the last three decades, and this paper tries to organize and present those in a meaningful and useful way, as will be seen in the next section. Some critical remarks about the directions taken by the researches on this subject are presented in the concluding section.

## Literature review

Vegetable pathologies may manifest in different parts of the plant. There are methods exploring visual cues present in almost all of those parts, like roots (Smith and Dickson 1991), kernels (Ahmad et al. 1999), fruits (Aleixos et al. 2002; Corkidi et al. 2005; López-García et al. 2010), stems and leaves. As commented before, this work concentrates in the latter two, particularly leaves.

This section is divided into three subsections according to the main purpose of the proposed methods. The subsections, in turn, are divided according to the main technical solution employed in the algorithm. A summarizing table containing information about the cultures considered and technical solutions adopted by each work is presented in the concluding section.

Some characteristics are shared by most methods presented in this section: the images are captured using consumer-level cameras in a controlled laboratory environment, and the format used for the images is RGB quantized with 8 bits. Therefore, unless stated otherwise, those are the conditions under which the described methods operate. Also, virtually all methods cited in this paper apply some kind of preprocessing to clean up the images, thus this information will be omitted from now on, unless some peculiarity warrants more detailing.

### Detection

Because the information gathered by applying image processing techniques often allows not only detecting the disease, but also estimating its severity, there are not many methods focused only in the detection problem. There are two main situations in which simple detection applies:

- Partial classification: when a disease has to be identified amidst several possible pathologies, it may be convenient to perform a partial classification, in which candidate regions are classified as being the result of the disease of interest or not, instead of applying a complete classification into any of the possible diseases. This is the case of the method by Abdullah et al. (2007), which is described in Section ‘Neural networks’.
- Real-time monitoring: in this case, the system continuously monitor the crops, and issues an alarm as soon as the disease of interest is detected in any of the plants. The papers by Sena Jr et al. (2003) and Story et al. (2010) fit into this context. Both proposals are also described in the following.

#### Neural networks

The method proposed by Abdullah et al. (2007) tries to discriminate a given disease (*corynespora*) from other pathologies that affect rubber tree leaves. The algorithm does not employ any kind of segmentation. Instead, Principal Component Analysis is applied directly to the RGB values of the pixels of a low resolution (15×15 pixels) image of the leaves. The first two principal components are then fed to a Multilayer Perceptron (MLP) Neural Network with one hidden layer, whose output reveals if the sample is infected by the disease of interest or not.

#### Thresholding

The method proposed by Sena Jr et al. (2003) aims to discriminate between maize plants affected by fall armyworm from healthy ones using digital images. They divided their algorithm into two main stages: image processing and image analysis. In the image processing stage, the image is transformed to a grey scale, thresholded and filtered to remove spurious artifacts. In the image analysis stage, the whole image is divided into 12 blocks. Blocks whose leaf area is less than 5% of the total area are discarded. For each remaining block, the number of connected objects, representing the diseased regions, is counted. The plant is considered diseased if this number is above a threshold, which, after empirical evaluation, was set to ten.

#### Dual-segmented regression analysis

Story et al. (2010) proposed a method for monitoring and early detection of calcium deficiency in lettuce. The first step of the algorithm is the plant segmentation by thresholding, so the canopy region is isolated. The outlines of the region of interest are applied back to the original image, in such a way only the area of interest is considered. From that, a number of color features (RGB and HSL) and texture features (from the gray-level co-occurrence matrix) are extracted. After that, the separation point identifying the onset of stress due to the calcium deficiency is calculated by identifying the mean difference between the treatment and control containers at each measured time for all features. Dual-segmented regression analysis is performed to identify where in time a change point was present between the nutrient-deficit group of plants and the healthy group of plants. The authors concluded arguing that their system can be used to monitor plants in greenhouses during the night, but more research is needed for its use during the day, when lighting conditions vary more intensely.

### Quantification

The methods presented in this section aim to quantify the severity of a given disease. Such a severity may be inferred either by the area of the leaves that are affected by the disease, or by how deeply rooted is the affection, which can be estimated by means of color and texture features. Most quantification algorithms include a segmentation step to isolate the symptoms, from which features can be extracted and properly processed in order to provide an estimate for the severity of the disease.

It is worth noting that the problem of determining the severity of a disease by analyzing and measuring its symptoms is difficult even if performed manually by one or more specialists, which have to pair the diagnosis guidelines with the symptoms as accurately as possible. As a result, the manual measurements will always contain some degree of subjectivity, which in turn means that references used to validate the automatic methods are not exactly “ground truth”. It is important to take this into consideration when assessing the performance of those methods.

The methods presented in the following are grouped according to the main strategies they employ to estimate the severity of the diseases.

#### Thresholding

One of the first methods to use digital image processing was proposed by Lindow and Webb (1983). The images were captured using an analog video camera, under a red light illumination to highlight the necrotic areas. Those images were later digitized and stored in a computer. The tests were performed using leaves from tomatoes, bracken fern, sycamore and California buckeye. The identification of the necrotic regions is done by a simple thresholding. The algorithm then apply a correction factor to compensate for pixel variations in the healthy parts of the leaves, so at least some of the pixels from healthy regions that were misclassified as part of the diseased areas can be reassigned to the correct set.

Price et al. (1993) compared visual and digital image-processing methods in quantifying the severity of coffee leaf rust. They tested two different imaging systems. In the first one, the images were captured by a black and white charge coupled device (CCD) camera, and in the second one, the images were captured with a color CCD camera. In both cases, the segmentation was performed by a simple thresholding. According to the authors, the image processing-based systems had better performance than visual evaluations, especially for cases with more severe symptoms. They also observed that the color imaging had greater potential in discriminating between rusted and non-rusted foliage.

The method proposed by Tucker and Chakraborty (1997) aims to quantify and identify diseases in sunflower and oat leaves. The first step of the algorithm is a segmentation whose threshold varies according to the disease being considered (blight or rust). The resulting pixels are connected into clusters representing the diseased regions. Depending on the characteristics of the lesions, they are classified into the appropriate category (type *a* or *b* in case of blight and by size in case of rust). The authors reported good results, but observed some errors due to inappropriate illumination during the capture of the images.

Martin and Rybicki (1998) proposed a method to quantify the symptoms caused by the maize streak virus. The thresholding scheme adopted by the authors was based on the strategy described by Lindow and Webb (1983) and briefly explained in the previous paragraph. The authors compared the results obtained by visual assessment, by using a commercial software package and by employing a custom system implemented by themselves. They concluded that the commercial and custom software packages had approximately the same performance, and that both computer-based methods achieved better accuracy and precision than the visual approach.

The method proposed by Skaloudova et al. (2006) measures the damage caused in leaves by spider mites. The algorithm is based on a two-stage thresholding. The first stage discriminates the leaf from the background, and the second stage separates damaged regions from healthy surface. The final estimate is given by the ratio between the number of pixels in damage regions divided by the total number of pixels of the leaf. The authors compared the results with two other methods based on the leaf damage index and chlorophyll fluorescence. They concluded that their method and the leaf damage index provided superior results when compared with the chlorophyll fluorescence.

In their work, Weizheng et al. (2008) presented a strategy to quantify lesions in soybean leaves. The algorithm is basically composed by a two-step thresholding. The first threshold aims to separate leaf from background. After that, the image containing only the leaf is converted to the HSI color space, and the Sobel operator is applied to identify the lesion edges. A second threshold is applied to the resulting Sobel gradient image. Finally, small objects in the binary image are discarded and holes enclosed by white pixels are filled. The resulting objects reveal the diseased regions.

Camargo and Smith (2009a) proposed a method to identify regions of leaves containing lesions caused by diseases. The tests were performed using leaves from a variety of plants, like bananas, maize, alfalfa, cotton and soybean. Their algorithm is based on two main operations. First, a color transformation to the HSV and I1I2I3 spaces is performed, from which only H and two modified versions of I3 are used in the subsequent steps. After that, a thresholding based on the histogram of intensities technique (Prewitt 1970) is applied in order to separate healthy and diseased regions. According to the authors, their approach was able to properly discriminate between diseased and healthy areas for a wide variety of conditions and species of plants.

The method proposed by Macedo-Cruz et al. (2011) aimed to quantify the damage caused by frost in oat crops. The images used by the authors were captured directly in the crop fields. The first step of the algorithm is the conversion from RGB to the L*a*b* representation. The authors employed three different thresholding strategies: Otsu’s method, Isodata algorithm, and fuzzy thresholding. Each strategy generates a threshold value for each color channel, which are combined by a simple average so a single threshold value is assigned to each channel. If necessary, the resulting partitions may be thresholded again, and so on, until some stopping criteria are met. The final resulting partitions give rise to a number of classes that, after properly labeled, reveal the extent of the damage suffered by the crops.

Lloret et al. (2011) proposed a system to monitor the health of vineyards. The images were captured by means of webcams scattered throughout the field. The main objective was to detected and quantify diseased leaves. Their system has five stages: 1) leaf size estimation, which is necessary due to the variation of the distance between the cameras and the plants; 2) thresholding, which separates diseased leaves and ground from healthy leaves using both the RGB and HSV color representations of the image; 3) a set of morphological operations, aiming to reduce noise without eliminating useful features; 4) a detection step, which aims to discriminate between ground and actual diseased leaves; 5) calculation of the ratio of diseased leaves. Depending on the value of this ratio, the system emits a warning that the plant requires some attention.

Patil and Bodhe (2011) proposed a method for assessing the severity of fungi-related disease in sugar cane leaves. The method performs two segmentations. The first one aims to separate the leaves from the rest of the scene, and is performed by means of a simple thresholding. In the second segmentation, the image is converted from the RGB to the HSI color space, and a binarization is applied in order to separate the diseased regions. The threshold for the binarization is calculated by the so-called triangle thresholding method, which is based on the gray-scale histogram of the image. The binary image is finally used to determine the ratio of the infection with respect to the entire leaf.

#### Color analysis

Boese et al. (2008) proposed a method to estimate the severity of eelgrass leaf injury, which can be caused by desiccation, wasting disease, and micro herbivory feeding. The first step of the algorithm is the unsupervised segmentation of the leaves into a number of classes (six to ten). In the following, an expert labels the classes into one of five possibilities (the three types of injuries, plus healthy tissue and background). After that, the quantification is just a matter of measuring the areas occupied by each of the injuries. According to the authors, their approach still have a number of problems that limit its utility, but it is an improvement over other approaches to quantify complex leaf injuries from multiple stressors.

The method proposed by Pagola et al. (2009) deals with the problem of quantifying nitrogen deficiency in barley leaves. They use some color channel manipulations in the RGB space and apply Principal Component Analysis (PCA) to obtain a measure for the “greenness” of the pixels. In order to aggregate the results of all pixels into a single estimate, the authors tested four strategies, whose main goal was to emphasize relevant regions and reduce the influence of the regions that are not photosinthetically active, like veins and leaf spots. The authors concluded that their method had high correlation with the largely adopted approach based on non-destructive hand-held chlorophyll meters.

Contreras-Medina et al. (2012) proposed a system to quantify five different types of symptoms in plant leaves. Their system is actually composed of five independent modules: 1) chlorosis algorithm, which combines the red and green components of the image in order to determine the yellowness of the leaf, which indicates the severity of the chlorosis; 2) necrosis algorithm, which uses the blue component to discriminate leaves from background, and the green component to identify and quantify the necrotic regions; 3) leaf deformation algorithm, which uses the blue component to segment the leaf and calculates the sphericity of the leaf as a measure for its deformation; 4) white spots algorithm, which applies a thresholding to the blue component of the image to estimate the area occupied by those spots; 5) mosaic algorithm, which uses the blue channel, a number of morphological operations and the Canny edge detector to identify and quantify the venations present in the leaf.

#### Fuzzy logic

In their paper, Sannakki et al. (2011) presented a method to quantify disease symptoms based on Fuzzy logic. The tests were performed using pomegranate leaves. The algorithm begins converting the images to the L*a*b* color space. The pixels are grouped into a number of classes through K-means clustering. According to the authors, one of the groups will correspond to the diseased areas, however the paper does not provide any information on how the correct group is identified. In the following, the program calculates the percentage of the leaf that is infected. Finally, a Fuzzy Inference System is employed for the final estimation of the disease rating. The details on how such a system is applied are also absent.

Sekulska-Nalewajko and Goclawski (2011) method aims to detect and quantify disease symptoms in pumpkin and cucumber leaves. The images used in the tests were captured using a flatbed scanner. The leaves were detached from the plants, treated and stained prior to the imaging. The authors used functions present in the Matlab toolboxes to implement their ideas. The first step of the algorithm is the isolation of the leaf by thresholding. In the following, the image is transformed from RGB to HSV color space. The brightness component (V) is discarded. Then, a Fuzzy c-means algorithm is applied in order to group the pixels into two main clusters, representing healthy and diseased regions. The authors argued that their approach is a better solution than using third-party packages which, according to them, require too many operations to achieve the desired results.

Zhou et al. (2011) proposed a method to evaluate the degree of hopper infestation in rice crops. The presence of rice plant-hoppers manifests more intensely in the stem, so that was the part of the plant focused by the authors. In the algorithm, after the regions of interest are extracted, fractal-dimension value features are extracted using the box-counting dimension method. These features are used to derive a regression model. Finally, a fuzzy C-means algorithm is used to classify the regions into one of four classes: no infestation, mild infestation, moderate infestation and severe infestation.

#### Knowledge-based system

The aim of the work by Boissard et al. (2008) was a little different from the others presented in this paper, as their method tries to quantify the number of whiteflies in rose leaves as part of an early pest detection system. The method employs two knowledge-based systems (KBS) to estimate the number of insects. The first system, the so-called classification KBS, takes the numerical results from some image processing operations, and interprets them into higher level concepts which, in turn, are explored to assist the algorithm to choose and retain only the regions containing insects. The second system, the so-called supervision KBS, selects the image processing tools to be applied, as well as the parameters to be used, in order to collect and feed the most meaningful information to the first system. According to the authors, their proposal had some problems, but it was a good addition to the efforts towards the automation of greenhouse operations.

#### Region growing

Pang et al. (2011) proposed a method to segment lesions caused by six types of diseases that affect maize crops. The algorithm begins by identifying all pixels for which the level of the red channel (R) is higher than the level of the green channel (G). According to the authors, those pixels are part of a diseased region in 98% of the cases. The connected regions are then identified and labeled. The second part of the algorithm tries to identify the pixels for which *R*<*G* that are actually part of the lesions. To do that, the algorithm takes the connected regions as seeds and applies a region growing technique to more accurately define the diseased regions. The termination condition for the growing procedure is given by the threshold values obtained by applying Otsu’s method to each connected region.

#### Third party image processing packages

Olmstead et al. (2001) compared two different methods (one visual and one computational) for quantifying powdery mildew infection in sweet cherry leaves. The images were captured using a flatbed scanner. The image analysis, which is basically the application of thresholding, was performed using the SigmaScan Pro (v. 4.0) software package. In order to generate a standard for comparison of the two methods, the fungi colonies were manually painted white and submitted to the image analysis, providing the reference values. According to the authors, the visual assessment provided far superior estimates in comparison with the computational one.

The method proposed by Berner and Paxson (2003) aimed at quantifying the symptoms in infected yellow starthistle. The images were captured using a flatbed scanner, and the images were analyzed by the SigmaScan Pro (v.5.0) software package. The operations applied to the image are simple: brightness and contrast adjustments, transformation to gray scale, and application of color overlays. Those overlays emphasize both diseased regions (pustules) and dark areas along venations, so a shape-based selection is carried out in order to keep only the diseased regions. Finally, the pustules are counted.

Moya et al. (2005) compared the results obtained by visual and image processing-based assessment of squash leaves infected with powdery mildew. They used a commercial software package, the ArcView GIS 3.2, to segment the leaf images into either five or ten classes. The assigned classes were then manually compared to the original images, and the regions corresponding to disease were properly labeled and measured. Finally, the severity of the disease was given by the number of selected pixels divided by the total number of pixels in the leaf. The authors compared these results to those obtained entirely manually. They also compared the results according to the type of device used for capturing the images (digital camera or scanner).

In their proposals, Bock et al. (2008,2009) aimed at quantifying the severity of the Foliar Citrus Canker in Grapefruit leaves. To perform the image analysis, the authors employed a package called Assess V1.0: Image Analysis Software for plant disease quantification (Lamari 2002). In their approach, the images are first converted to the HSI format, and then thresholded to separate the diseased parts from the rest of the scene. The value of the threshold was initially tuned manually by visually comparing the resulting segmentation with the actual image. After the ideal segmentation is achieved, estimating the severity is just a matter of calculating the healthy and diseased areas and finding their ratio. The authors later tried to automate the thresholding process, achieving some mixed results due to tone and lighting variations that prevent fixed thresholds to be valid in all cases.

Goodwin and Hsiang (2010) and Wijekoon et al. (2008) used a freely available software called Scion Image to quantify fungal infection in leaves of lilies-of-the-valley, apple trees, phlox and golden rod. The images were captured both in laboratory and *in situ*, using flatbed scanners for detached leaves and consumer level digital cameras for attached leaves. The use of the Scion software was almost entirely based upon the method proposed by Murakami (2005), in which the color of a targeted area is manually adjusted in order to maximize the discrimination between healthy and diseased surfaces. The symptoms of several fungal diseases were tested, like powdery mildew, rust, anthracnose and scab.

The Assess software (v. 2.0) was used by Coninck et al. (2012) to determine the severity of Cercospora leaf spot (CLS) disease in sugar beet breeding. Their approach was related to that used by Bock et al. (2009), with the images being converted to the HSI representation and with a properly threshold being determined by means of practical experiments. The main purpose of the authors was not to develop a novel method for disease symptom quantification, but to compare the accuracy of three very different ways of estimating the disease severity: visual assessment, real-time Polymerase Chain Reaction (PCR) and image processing. The authors concluded stating that the use of both image analysis and real-time PCR had the potential to increase accuracy and sensitivity of assessments of CLS in sugar beet, while reducing bias in the evaluations.

The software package ImageJ was used by Peressotti et al. (2011) to quantify grapevine downy mildew sporulation. The authors wrote a macro for ImageJ, which properly adjusts color balance and contrast prior to presenting the image to the user. After that, the user can test several different values of threshold to segment the image, until a satisfactory result is achieved. The authors reported good correlation between the results obtained by their method and by visual assessment.

### Classification

The classification methods can be seen as extensions of the detection methods, but instead of trying to detect only one specific disease amidst different conditions and symptoms, these ones try to identify and label whichever pathology that is affecting the plant. As in the case of quantification, classification methods almost always include a segmentation step, which is normally followed by the extraction of a number of features that will feed some kind of classifier. The methods presented in the following are grouped according to the kind of classification strategy employed.

#### Neural networks

A very early attempt to monitor plant health was carried out by Hetzroni et al. (1994). Their system tried to identify iron, zinc and nitrogen deficiencies by monitoring lettuce leaves. The capture of the images was done by an analog video camera, and only afterwards the images would be digitized. The first step of the proposed algorithm is the segmentation of the images into leaf and background. In the following a number of size and color features are extracted from both the RGB and HSI representations of the image. Those parameters are finally fed to neural networks and statistical classifiers, which are used to determine the plant condition.

Pydipati et al. (2005) compared two different approaches to detect and classify three types of citrus diseases. The authors collected 39 texture features, and created four different subsets of those features to be used in two different classification approaches. The first approach was based on a Mahalanobis minimum distance classifier, using the nearest neighbor principle. The second approach used radial basis functions (RBF) neural network classifiers trained with the backpropagation algorithm. According to the authors, both classification approaches performed equally well when using the best of the four subsets, which contained ten hue and saturation texture features.

Huang (2007) proposed a method to detect and classify three different types of diseases that affect Phalaenopsis orchid seedlings. The segmentation procedure adopted by the author is significantly more sophisticated than those found in other papers, and is composed by four steps: removal of the plant vessel using a Bayes classifier, equalization of the image using an exponential transform, a rough estimation for the location of the diseased region, and equalization of the sub-image centered at that rough location. A number of color and texture features are then extracted from the gray level co-occurrence matrix (Haralick et al. 1973). Finally, those features are submitted to an MLP artificial neural network with one hidden layer, which performs the final classification.

Sanyal et al. (2007) tackled the problem of detecting and classifying six types of mineral deficiencies in rice crops. First, the algorithm extracts a number of texture and color features. Each kind of feature (texture and color) is submitted to its own specific MLP neural network. Both networks have one hidden layer, but the number of neurons in the hidden layer is different (40 for texture and 70 for color). The results returned by both networks are then combined, yielding the final classification. A very similar approach is used by the same authors in another paper (Sanyal and Patel 2008), but in this case the objective is to identify two kinds of diseases (blast and brown spots) that affect rice crops.

The method proposed by Al Bashish et al. (2010) tries to identify five different plant diseases. The authors did not specify the species of plants used in the tests, and the images were captured *in situ*. After a preprocessing stage to clean up the image, a K-means clustering algorithm is applied in order to divide the image into four clusters. According to the authors, at least one of the clusters must correspond to one of the diseases. After that, for each cluster a number of color and texture features are extracted by means of the so-called Color Co-Occurrence Method, which operates with images in the HSI format. Those features are fed to a MLP Neural Network with ten hidden layers, which performs the final classification.

Kai et al. (2011) proposed a method to identify three types of diseases in maize leaves. First, the images are converted to the YCbCr color representation. Apparently, some rules are applied during the thresholding in order to properly segment the diseased regions. However, due to a lack of clarity, it is not possible to infer exactly how this is done. The authors then extract a number of texture features from the gray level co-occurrence matrix. Finally, the features are submitted to an MLP neural network with one hidden layer.

Wang et al. (2012) proposed a method to discriminate between pairs of diseases in wheat and grapevines. The images are segmented by a K-means algorithm, and then 50 color, shape and texture features are extracted. For the purpose of classification, the authors tested four different kinds of neural networks: Multilayer Perceptron, Radial Basis Function, Generalized Regression, and Probabilistic. The authors reported good results for all kinds of neural networks.

#### Suport vector machines

Meunkaewjinda et al. (2008) proposed a method to identify and classify diseases that affect grapevines. The method uses several color representations (HSI, L*a*b*, UVL and YCbCr) throughout its execution. The separation between leaves and background is performed by an MLP neural network, which is coupled with a color library built a priori by means of an unsupervised self organizing map (SOM). The colors present on the leaves are then clustered by means of an unsupervised and untrained self-organizing map. A genetic algorithm determines the number of clusters to be adopted in each case. Diseased and healthy regions are then separated by a Support Vector Machine (SVM). After some additional manipulations, the segmented image is submitted to a multiclass SVM, which performs the classification into either scab, rust, or no disease.

Youwen et al. (2008) proposed a method to identify two diseases that can manifest in cucumber leaves. The segmentation into healthy and diseased regions is achieved using a statistic pattern recognition approach. In the following, some color, shape and texture features are extracted. Those features feed an SVM, which performs the final classification. The authors stated that the results provided by the SVM are far better than those achieved using neural networks.

The system proposed by Yao et al. (2009) aimed to identify and classify three types of diseases that affect rice crops. The algorithm first applies a particular color transformation to the original RGB image, resulting in two channels (*y* 1 and *y* 2). Then, the image is segmented by Otsu’s method, after which the diseased regions are isolated. Color, shape and texture features are extracted, the latter one from the HSV color space. Finally, the features are submitted to a Support Vector Machine, which performs the final classification.

The method proposed by Camargo and Smith (2009b) tries to identify three different kinds of diseases that affect cotton plants. The authors used images not only of leaves, but also of fruits and stems. The segmentation of the image is performed using a technique developed by the authors (Camargo and Smith 2009a), which was described earlier in this paper (Section ‘Thresholding’). After that, a number of features is extracted from the diseased regions. Those features are then used to feed an SVM. The one-against-one method (Hsu and Lin 2002) was used to allow the SVM to deal with multiple classes. The authors concluded that the texture features have the best discrimination potential.

Jian and Wei (2010) proposed a method to recognize three kinds of cucumber leaf diseases. As in most approaches, the separation between healthy and diseased regions is made by a simple thresholding procedure. In the following, a variety of color, shape and texture features are extracted. Those features are submitted to an SVM with Radial Basis Function (RBF) as kernel, which performs the final classification.

#### Fuzzy classifier

The method proposed by Hairuddin et al. (2011) tries to identify four different nutritional deficiencies in oil palm plants. The image is segmented according to color similarities, but the authors did not provide any detail on how this is done. After the segmentation, a number of color and texture features are extracted and submitted to a fuzzy classifier which, instead of outputting the deficiencies themselves, reveals the amounts of fertilizers that should be used to correct those deficiencies. Unfortunately, the technical details provided in this paper are superficial, making it difficult to reach a clear understanding about the approach adopted by the authors.

Xu et al. (2011) proposed a method to detect nitrogen and potassium deficiencies in tomato plants. The algorithm begins extracting a number of features from the color image. The color features are all based on the b* component of the L*a*b* color space. The texture features are extracted using three different methods: difference operators, Fourier transform and Wavelet packet decomposition. The selection and combination of the features was carried out by means of a genetic algorithm. Finally, the optimized combination of features is used as the input of a fuzzy K-nearest neighbor classifier, which is responsible for the final identification.

#### Feature-based rules

In their two papers, Kurniawati et al. ([Bibr CR25][Bibr CR26]) proposed a method to identify and label three different kinds of diseases that affect paddy crops. As in many other methods, the segmentation of healthy and diseased regions is performed by means of thresholding. The authors tested two kinds of thresholding, Otsu’s and local entropy, with the best results being achieved by the latter one. Afterwards, a number of shape and color features are extracted. Those features are the basis for a set of rules that determine the disease that best fits the characteristics of the selected region.

Zhang (2010) proposed a method for identifying and classifying lesions in citrus leaves. The method is mostly based on two sets of features. The first set was selected having as main goal to separate lesions from the rest of the scene, which is achieved by setting thresholds to each feature and applying a weighted voting scheme. The second set aims to provide as much information as possible about the lesions, so a discrimination between diseases becomes possible. The final classification is, again, achieved by means of feature thresholds and a weighted voting system. A more detailed version of (Zhang 2010) can be found in (Zhang and Meng 2011).

#### Color analysis

The method proposed by Wiwart et al. (2009) aims to detect and discriminate among four types of mineral deficiencies (nitrogen, phosphorus, potassium and magnesium). The tests were performed using faba bean, pea and yellow lupine leaves. Prior to the color analysis, the images are converted to the HSI and L*a*b* color spaces. The presence or absence of the deficiencies is then determined by the color differences between healthy leaves and the leaves under test. Those differences are quantified by Euclidean distances calculated in both color spaces.

Pugoy and Mariano (2011) proposed a system to identify two different types of diseases that attack rice leaves. The algorithm first converts the image from RGB to HSI color space. The K-means technique is applied to cluster the pixels into a number of groups. Those groups are then compared to a library that relates colors to the respective diseases. This comparison results in values that indicate the likelihood of each region being affected by each of the diseases.

#### Self organizing maps

The method proposed by Phadikar and Sil (2008) detects and differentiates two diseases that affect rice crops, blast and brown spots. First, the image is converted to the HSI color space. Then, a entropy-based thresholding is used to segment the image. An edge detector is applied to the segmented image, and the intensity of the green components is used to detect the spots. Each region containing each detected spot is then resized by interpolation, so all regions have a size of 80×100 pixels. The pixel values (gray scale) are finally fed to a self organizing map (SOM), which performs the final classification.

#### Discriminant analysis

Pydipati et al. (2006) method aims to detect and classify three different types of citrus diseases. The method relies heavily on the color co-occurrence method (CCM), which, in turn, was developed through the use of spatial gray-level dependence matrices (SGDM’s) (Shearer and Holmes 1990). The resulting CCM matrices, which are generated from the HSI color representation of the images, are used to extract 39 texture features. The number of features was then reduced by means of a redundancy reduction procedure. The authors observed that the elimination of intensity features improved the results, as hue and saturation features are more robust to ambient light variations than the former ones. The final classification was performed using discriminant analysis.

#### Membership function

Anthonys and Wickramarachchi (2009) proposed a method to discriminate among three different diseases that attack paddy plants. The image is segmented by a thresholding procedure – the grayscale version of the image used in such a procedure is obtained after assigning different weights to each component of its RGB representation. The resulting images, containing only the regions supposedly containing the symptoms of the diseases, are then converted to the L*a*b* format, and a number of color and shape features are extracted. The values of those features are compared to some reference value intervals stored in a lookup table by means of the so-called Membership Function, which outputs a single similarity score for each possible disease. The highest score determines the disease affecting the plant.

## Discussion

Table 1 shows an overview of all methods presented in this paper, together with the type of plant considered in each research and the main technical solution used in the algorithm.

**Table 1:** **Summarization of the proposals**

Proposal	Type	Main tool	Culture
Abdullah et al. (2007)	Detection	Neural networks	Rubber tree
Al Bashish et al. (2010)	Classification	Neural networks	N/A
Anthonys and Wickramarachchi (2009)	Classification	Membership function	Rice
Berner and Paxson (2003)	Quantification	3rd party package	Yellow starthistle
Bock et al. (2008)	Quantification	3rd party package	Grapefruit
Bock et al. (2009)	Quantification	3rd party package	Grapefruit
Boese et al. (2008)	Quantification	Color analysis	Eelgrass
Boissard et al. (2008)	Quantification	Knowledge-based	Roses
Camargo and Smith (2009a)	Quantification	Thresholding	Banana, maize, soy, alfalfa, cotton
Camargo and Smith (2009b)	Classification	SVM	Cotton
Coninck et al. (2012)	Quantification	3rd party package	Sugar beet
Contreras-Medina et al. (2012)	Quantification	Color analysis	Pumpkin, pepper, bean
Goodwin and Hsiang (2010)	Quantification	3rd party package	Lilies, apple tree
			phlox and golden rod
Hairuddin et al. (2011)	Classification	Fuzzy logic	Oil palm
Hetzroni et al. (1994)	Classification	Neural networks	Lettuce
Huang (2007)	Classification	Neural networks	Orchid
Jian and Wei (2010)	Classification	SVM	Cucumber
Kai et al. (2011)	Classification	Neural networks	Maize
Kurniawati et al. (2009a)	Classification	Feature-based rules	Rice
Kurniawati et al. (2009b)	Classification	Feature-based rules	Rice
Lindow and Webb (1983)	Quantification	Thresholding	Sycamore, fern, tomato, buckeye
Lloret et al. (2011)	Quantification	Thresholding	Grapes
Macedo-Cruz et al. (2011)	Quantification	Thresholding	Oat
Martin and Rybicki (1998)	Quantification	Thresholding	Maize
Meunkaewjinda et al. (2008)	Classification	SVM	Grapes
Moya et al. (2005)	Quantification	3rd party package	Squash
Olmstead et al. (2001)	Quantification	3rd party package	Cherry
Pagola et al. (2009)	Quantification	Color analysis	Barley
Pang et al. (2011)	Quantification	Region growing	Maize
Patil and Bodhe (2011)	Quantification	Thresholding	Sugar cane
Peressotti et al. (2011)	Quantification	3rd party package	Grapes
Phadikar and Sil (2008)	Classification	Self organizing maps	Rice
Price et al. (1993)	Quantification	Thresholding	Coffee
Pugoy and Mariano (2011)	Classification	Color analysis	Rice
Pydipati et al. (2005)	Classification	Neural networks	Orange
Pydipati et al. (2006)	Classification	Self organizing maps	Orange
Sannakki et al. (2011)	Quantification	Fuzzy logic	Pomegranate
Sanyal et al. (2007)	Classification	Neural networks	Rice
Sanyal and Patel (2008)	Classification	Neural networks	Rice
Sekulska-Nalewajko and Goclawski (2011)	Quantification	Fuzzy logic	Pumpkin, cucumber
Sena Jr et al. (2003)	Detection	Thresholding	Maize
Skaloudova et al. (2006)	Quantification	Thresholding	Bean
Story et al. (2010)	Detection	Dual-segmented regression analysis	Lettuce
Tucker and Chakraborty (1997)	Quantification	Thresholding	Oat, sunflower
Wang et al. (2012)	Classification	Neural networks	Grapes, wheat
Weizheng et al. (2008)	Quantification	Thresholding	Soy
Wijekoon et al. (2008)	Quantification	3rd party package	Lilies, apple tree
			phlox and golden rod
Wiwart et al. (2009)	Classification	Color analysis	Faba beans, pea, yellow lupine
Xu et al. (2011)	Classification	Fuzzy logic	tomato
Yao et al. (2009)	Classification	SVM	Rice
Youwen et al. (2008)	Classification	SVM	Cucumber
Zhang (2010)	Classification	Feature-based rules	Orange
Zhang and Meng (2011)	Classification	Feature-based rules	Orange
Zhou et al. (2011)	Quantification	Fuzzy logic	Rice
Xu et al. (2011)	Classification	Fuzzy logic	Tomato
Yao et al. (2009)	Classification	SVM	Rice
Youwen et al. (2008)	Classification	SVM	Cucumber
Zhang (2010)	Classification	Feature-based rules	Orange
Zhang and Meng (2011)	Classification	Feature-based rules	Orange
Zhou et al. (2011)	Quantification	Fuzzy logic	Rice

Despite the importance of the subject of identifying plant diseases using digital image processing, and although this has been studied for at least 30 years, the advances achieved seem to be a little timid. Some facts lead to this conclusion:

- Methods are too specific. The ideal method would be able to identify any disease in any kind of plant. Evidently, this is unfeasible given the current technological level. However, many of the methods that are being proposed not only are able to deal with only one species of plant, but those plants need to be at a certain growth stage in order to the algorithm to be effective. That is acceptable if the disease only attacks the plant in that specific stage, but it is very limiting otherwise. Many of the papers do not state this kind of information explicitly, but if their training and test sets include only images of a certain growth stage, which is often the case, the validity of the results cannot be extended to other stages.
- Operation conditions are too strict. Many images used to develop new methods are collected under very strict conditions of lighting, angle of capture, distance between object and capture device, among others. This is a common practice and is perfectly acceptable in the early stages of research. However, in most real world applications, those conditions are almost impossible to be enforced, especially if the analysis is expected to be carried out in a non-destructive way. Thus, it is a problem that many studies never get to the point of testing and upgrading the method to deal with more realistic conditions, because this limits their scope greatly.
- Lack of technical knowledge about more sophisticated technical tools. The simplest solution for a problem is usually the preferable one. In the case of image processing, some problems can be solved by using only morphological mathematical operations, which are easy to implement and understand. However, more complex problems often demand more sophisticated approaches. Techniques like neural networks, genetic algorithms and support vector machines can be very powerful if properly applied. Unfortunately, that is often not the case. In many cases, it seems that the use of those techniques is founded more in the hype they generate in the scientific community than in their technical appropriateness with respect to the problem at hand. As a result, problems like overfitting, overtraining, undersized sample sets, sample sets with low representativeness, bias, among others, seem to be a widespread plague. Those problems, although easily identifiable by a knowledgeable individual on the topic, seem to go widely overlooked by the authors, probably due to the lack of knowledge about the tools they are employing. The result is a whole group of technically flawed solutions.

Evidently, there are some high quality manuscripts in which the authors rigorously take into account most factors that could harm the validity of their results, but unfortunately those still seem to be the exception, not the rule. As a result, the technology evolves slower than it could. The underlying conclusion is that the authors should spend a little more time learning about the tools they intend to use. A better understand about the concepts behind those tools can potentially lead to more solid results and to less time wasted, improving the overall quality of the literature of the area.

## Conclusion

The wide-ranging variety of applications on the subject of counting objects in digital images makes it difficult for someone to prospect all possible useful ideas present in the literature, which can cause potential solutions for problematic issues to be missed. In this context, this paper tried to present a comprehensive survey on the subject, aiming at being a starting point for those conducting research on the issue. Due to the large number of references, the descriptions are short, providing a quick overview of the ideas underlying each of the solutions. It is important to highlight that the work on the subject is not limited to what was shown here. Many papers on the subject could not be included in order to keep the paper length under control – the papers were selected as to consider the largest number of different problems as possible. Thus, if the reader wishes to attain a more complete understanding on a given application or problem, he/she can refer to the bibliographies of the respective articles.
